# *Staphylococcus aureus* Epidemiology in Wildlife: A Systematic Review

**DOI:** 10.3390/antibiotics9020089

**Published:** 2020-02-18

**Authors:** Christina J. Heaton, Gracen R. Gerbig, Lucas D. Sensius, Vishwash Patel, Tara C. Smith

**Affiliations:** Kent State University, College of Public Health, Kent, OH 44240, USA; cheaton5@kent.edu (C.J.H.); ggerbig@kent.edu (G.R.G.); sensiusl@gmail.com (L.D.S.); vpatel31@kent.edu (V.P.)

**Keywords:** antibiotic resistance, molecular typing, environment, zoonosis

## Abstract

*Staphylococcus aureus* is a common bacterial colonizer of humans and a variety of animal species. Many strains have zoonotic potential, moving between humans and animals, including livestock, pets, and wildlife. We examined publications reporting on *S. aureus* presence in a variety of wildlife species in order to more cohesively review distribution of strains and antibiotic resistance in wildlife. Fifty-one studies were included in the final qualitative synthesis. The most common types documented included ST398, ST425, ST1, ST133, ST130, and ST15. A mix of methicillin-resistant and methicillin-susceptible strains were noted. A number of molecular types were identified that were likely to be found in wildlife species, including those that are commonly found in humans or other animal species (including livestock). Additional research should include follow-up in geographic areas that are under-sampled in this study, which is dominated by European studies.

## 1. Introduction

*Staphylococcus aureus* is a common commensal bacterium that lives within the nares, throat, and on the skin of humans and a wide variety of animal species. *S. aureus* can spread via person-to-person contact (directly or mediated by fomites) and can be transmitted zoonotically via direct contact with animals or animal products, including raw meats [1]. It can also be maintained in the environment in manure, water, or the air [2].

Because of its frequency in various environments and species, it is critical to understand movement within and between communities. *S. aureus* is frequently resistant to one or more classes of antibiotics, and the continued spread of methicillin-resistant *S. aureus* (MRSA) over the past several decades in both human and animal species has increased the risk of acquiring a resistant infection that makes treatment more difficult and costly [3].

The epidemiology of MRSA in particular has changed over the past 30 years [4]. Once primarily a hospital-associated pathogen, the rise of novel strains of MRSA in the 1990s outside of the nosocomial environment led to the recognition of “community-associated MRSA” (CA-MRSA), in contrast with the historic hospital-associated (HA-MRSA) strains [5]. In the mid-2000s, a third genre of MRSA was recognized, as colonization and infection of livestock and livestock workers led to the designation of livestock-associated MRSA (LA-MRSA) [6]. It should be noted that we know less about the changes in methicillin-susceptible *S. aureus* during this period (MSSA), as the bulk of surveillance is dedicated to MRSA and does not always capture MSSA epidemiology.

Wildlife are a special case and often under-studied in the epidemiology of antibiotic resistance in the community and environment. Wildlife can act as reservoirs for intrinsic resistance elements or organisms (those that are naturally occurring in the environment, including environmental bacteria and fungi living in soil and water) and may also be exposed to resistant organisms or resistance genes in the environment amplified via human activity. This may be via treated humans who excrete resistant bacteria, antimicrobials that eventually end up in sewage effluent dispersed into the environment, or from sludge or waste from humans or livestock dispersed on fields as fertilizer. This may lead to further dissemination into streams, rivers, and larger waterways and also allow for airborne transmission of dried materials. Resistance may also be generated in the environment due to spraying of antibiotics on citrus groves and other plants [7] as well as by use in aquaculture [8].

*S. aureus* is a commensal organism that is able to effectively colonize a wide variety of host species, including many mammalian species but also birds and fish. As such, animals besides human have the potential to harbor novel strains of *S. aureus*, which could enter the human population, or conversely, humans may also transmit strains of *S. aureus* to other animal species [9], which can then acquire additional resistance genes.

The clearest evidence of zoonotic transmission of *S. aureus* has been in livestock. Isolates of clonal complex 398 appear to have originated as a human-adapted lineage but were transmitted to livestock including pigs and cattle and have become both more antibiotic-resistant (including MRSA strains) and have also typically lost some human virulence factors [9]. A similar situation appears to have happened with CC5 in poultry [10]. Recent research also suggests an emerging lineage in humans, strains of CC130, originated in cattle, and typically carry a novel methicillin resistance gene (*mecC*, originally called *mecA*_LGA251_) [11]. The role other animals may play in such cross-species transmission is less defined. Systematic collection and molecular typing of *S. aureus* from animal species has not been a priority; as such, cross-species transmission events from such animals to humans or wildlife to better-studied animal populations (including livestock and poultry) have likely been missed. This review examines the epidemiology of *S. aureus* in wildlife, including molecular typing and antibiotic resistance data where available.

## 2. Results

### 2.1. Search Results

**Figure 1:**
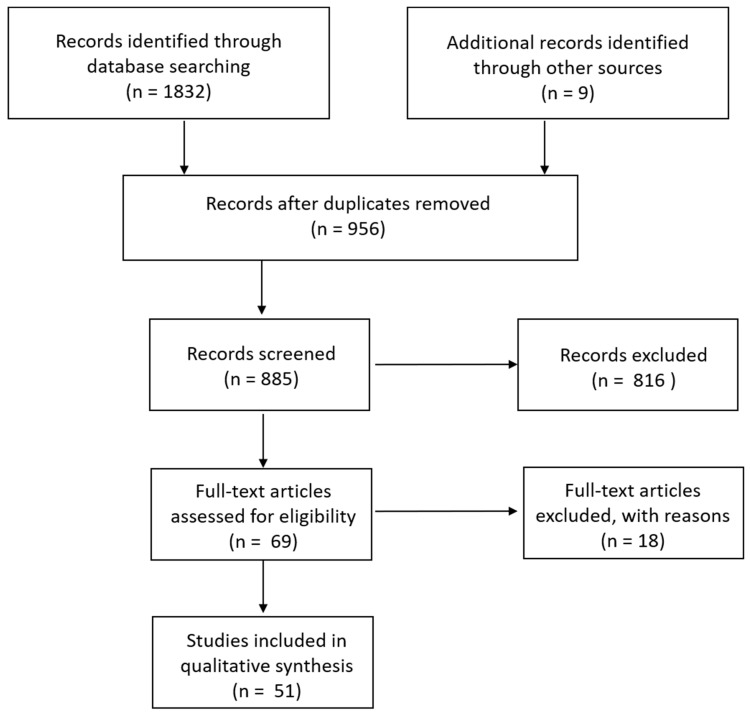
Schematic of search strategy.

Searching within Pubmed resulted in 856 hits, Web of Science in 58, and peer-reviewed materials within ProQuest in 918, for a total of 1832 publications. Upon searching references for additional studies that had been missed by our search terms, another nine were added. Titles and abstracts were examined to exclude duplicates (956); this left 885 remaining. Additional publications were excluded if they used animals only as an experimental model rather than examined epidemiology in wild species (such as rats, guinea pigs, and rabbits) and those that only mentioned *S. aureus* within the discussion or otherwise rather than consisted of a study focused on *S. aureus* epidemiology in wildlife. This left 69 for analysis (Figure 1).

### 2.2. Full-Text Articles Excluded

Eighteen studies were included within the initial analysis but excluded from further analysis Table 1 due to lack of detail reported regarding the *S. aureus* detected. These studies included the identification of *S. aureus* in a white ibis in Egypt [12], captive bustards in the United Arab Emirates [13], a peregrine falcon (*Falco peregrinus*) in Spain [14], from “free living insectivores” including the common shrew, lesser shrew, bank vole, root vole, and field mouse [15]; *S. aureus* was reported in this study but were not typed nor reported which species were positive. In Brazil, an opossum with mastitis was described but neither molecular typing nor antibiotic resistance phenotype was provided [16]. Similarly, a systemic *S. aureus* infection in a raccoon was also reported but not further characterized [17]. A 2013 study suggests that *S. aureus* infection is an important skin disease of red squirrels (*Sciurus vulgaris*) in Great Britain [18], and a Canadian publication determined that *S. aureus* was a common organism found in bite wounds from Norway and Black rats (*Rattus norvegicus* and *Rattus rattus*, respectively) [19], but no details were provided in either paper. *S. aureus* was also found along with other *mecA*-positive staphylococci in foxes in the United Kingdom, but samples were not typed [20]. *S. aureus*-positive Spanish Ibex (*Capra pyrenaica hispanica*) were identified in Spain but not typed or examined phenotypically [21]. A black rhinocerous (*Diceros bicornis*) in Kenya was reported to have an *S. aureus* infection (a possible cause of mortality) but also lacks details [22]. Bighorn sheep (*Ovis canadensis nelson* and *Ovis canadensis mexicana*) in Arizona were found to be colonized with *S. aureus* [23], but it was not characterized. *S. aureus* was identified in fecal samples taken from red deer in Poland [24] and from fecal samples from slaughtered reindeer in Finland and Norway [25] but was not further characterized. Finally, *S. aureus* was isolated from bottlenose dolphins (*Tursiops truncatus*) in the southeastern United States, but it was not further characterized [26,27]. Multiple zoo animals in Belgium were tested for MRSA, but no positive samples were reported [28]. Schaumburg [29] was not included in Table 1 because species are not specific (monkey, goat, etc. rather than exact species types) but demonstrates some sharing of *spa* types between humans, domestic animals, and wildlife (more for the former than the latter).

### 2.3. Molecular Types

**Figure 2:**
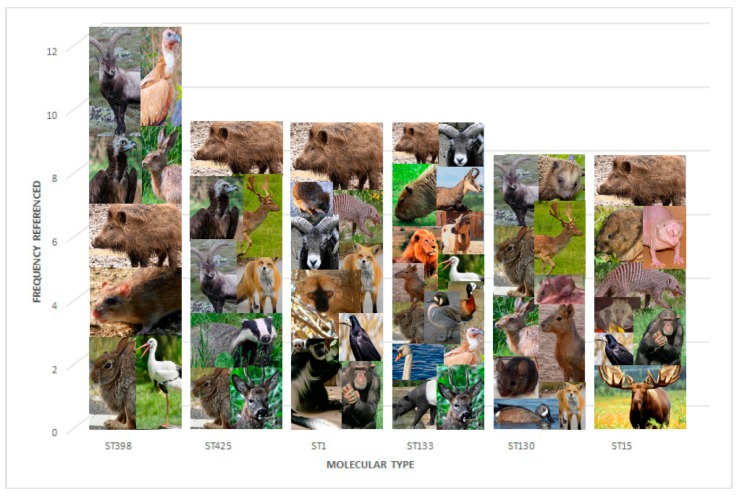
Molecular types identified in multiple papers and associated species. Sources of photos are included in Appendix A.

An examination of the molecular types found in wildlife demonstrates an extensive diversity of types of *S. aureus*. However, some broad conclusions can be suggested. Though comparisons across publications are difficult due to divergent methodology of sampling, testing, and geography, Figure 2 illustrates the most common molecular types, according to the total count publications identifying them. These molecular types include a mix of human pandemic types (ST5, ST8, ST1, ST30, ST22) [82] and molecular types that have been more commonly described in animals or at the animal–human interface (ST398, ST130, ST133, ST425) [83,84].

## 3. Discussion

This review demonstrates a significant amount of diversity in *Staphylococcus aureus* sampled from a wide variety of wildlife species across several continents. Populations of *S. aureus* present in wildlife may serve as reservoirs that could be transmitted to nearby domestic livestock or poultry or directly or indirectly to humans. Such a reservoir of *S. aureus* in the environment may also contribute to the exchange of antibiotic resistance or virulence genes among human or animal *S. aureus*, potentially leading to novel strains.

The continuing encroachment of humans into animal spaces due to agriculture, deforestation, climate change can lead to “spillovers” of pathogens from one species to another [85]. Most commonly we examine this with wildlife as a reservoir and humans as the affected species (e.g., Ebola, Nipah, MERS, SARS). However, transmission may also occur in reverse, with humans seeding wildlife with pathogens [86,87]. In the case of *S. aureus*, it appears both may be occurring, as has been previously documented among livestock [9]. In the case of antibiotic-resistant pathogens, such bidirectional transmission may be direct, via contact between human and animal species. More likely in the case of wildlife species, transmission may be indirect, such as via environmental reservoirs of pathogens including water sources, soil, exposure to manure, air, and contact with contaminated fomites [88,89]. Transmission may also occur due to consumption of meat products contaminated with *S. aureus,* but sampling wildlife meat products is exceedingly difficult and has not been done in a systematic manner. Meat products from livestock are a potential way that livestock-associated strains of *S. aureus* may spread from farms to communities [1,89], but the impact of meat from wildlife sources (including various deer species and wild boar) which may play a role in transmission of *S. aureus* bacteria or resistant genes is less clear.

While few studies reviewed here examine the environment and wildlife at the same time, a study by Porrero et al. [90] found *mec*C-positive *S. aureus* in river water after the area had been found to be positive for ST425-*mec*C in wild boar and fallow deer at the same location [54], suggesting a shared source of exposure or transmission between the various animal species and/or the environment.

Indeed, ST425 is a dominant molecular type that was found in wildlife papers. It does not appear to have a particular host specificity, with isolation reported from mammals including rabbits [35], boar [35,40,53,55,57,58], red deer [35,40,54], and roe deer [40,44] and from vultures [75] (see also Table 1 and Figure 1); these were found exclusively in European countries. The significance of this finding is currently unknown. ST425 isolates are known to be zoonotic, and have been described as a human colonizer as well, and its ability to cross species barriers may facilitate transmission of resistance genes, including *mec*C [11]. Other key molecular types present in a wide variety of species included ST398 in Norway rats [30,31,32,33,34], brown hare [40], boar [53,54,56], red deer [53,54], Iberian ibex [54], vulture [75], white stork [76], Eurasian griffon vulture [54], and Canada goose [79] and ST130/CC130, found in a rabbit [35], hedgehog [37,38,40], wood mouse [41], brown rat [40], yellow-necked mouse [42], house mouse [42], brown hare [37,40], mara [48,49], red fox [40], boar [35,55], red deer [35,59], Iberian ibex [53], fallow deer [40], and blue-winged teal [44]. The latter includes a large number of small animals and rodents, suggesting these may be an important reservoir in addition to livestock [91], and ST398 is a known colonizer of humans, particularly those with livestock contact [84]. Colonization may result in transmission of antibiotic resistance genes between species, while ST398 is also capable of causing a wide range of infections in humans [92].

Interestingly, bats and non-human primates seem to have little overlap with other animal strains. Bat molecular types consisted primarily of newly identified *spa* and/or MLST, though ST15 was reported twice—in a straw-colored fruit bat in Nigeria, and a captive Egyptian fruit bat sampled in Denmark [49,50].

For primate *S. aureus*, the papers reviewed here represent a mix of primates raised in captivity (including zoos and research facilities) and those sampled in sanctuaries and parks. As such, intensity of contact with humans who may be carrying typical human strains of *S. aureus* varies widely, and the importance of cross-species transmission remains in debate. Human-to-primate transmission was suggested in a study of wild primates MRSA in Nepal [63] and primates in Gabon [70]. The reverse was suggested by examination of an ST3268 strain found in macaques in primate research facilities in Singapore [68] and the United States [66,67]; this molecular type was also found in macaques in a New York research facility [65], suggesting the need for screening of animals prior to export/import. While most reports suggest preponderance of primate-associated strains, testing in a Texas facility found that their animals were colonized primarily with USA300/ST8 strains, which are common in humans and suggestive of human-to-animal transmission. However, workers at the facility were not tested for carriage [72].

Though *S. aureus* strains were typically taken as colonizers from healthy animals, several primates were actively infected with *S. aureus*. A gorilla in a primate center in Gabon was found to have a large lesion on his back; the gorilla died suddenly, and autopsy also found *S. aureus* in tissue samples; all were *spa* type t148 [70]. Though this is a human-associated strain, sampling of caretakers did not show any colonized humans involved in the animal’s care. In the Washington state facility, *S. aureus* was cultured from the wounds of two macaques, but both were likely primate strains (t15469/ST3268 and t13638/ST3268) [66]. Another publication from Korea documented a macaque with an acute necrotic lesion caused by MRSA, but molecular typing was not carried out [64].

How may exposure to human pathogens, including *S. aureus*, in great ape populations affect release of them back into wild from captivity? This is addressed in several publications, suggesting that primates from captivity may pose a risk to their wild brethren [74] due to carriage of organisms such as drug-resistant *S. aureus*. Others argue release still should be possible but caregivers should be screened, and those positive for *S. aureus* carriage should not have contact with infant apes, and post-release monitoring of animals should include screening for this bacterium [93]. This may be difficult given the high level of carriage found in wild primates (up to 100% of chimpanzees tested and 72% of lemurs) [73].

While most studies examined asymptomatic colonization of wildlife, in some reports, such as those from captive zoo animals ([44]), a number of clinical infections could be examined. These infections included abscesses, bacteremia, bite wounds, and dermatitis, among other conditions. Common animal-associated lineages were found, including CC130, CC133, and bacteremia caused by CC398 in an African wildcat. There was considerable diversity among the infection isolates, though a few did share spa or ST/CC types including two cases of t208/ST49/CC49 infections in red squirrels, two cases of t1166/ST3269/CC133 infections in a black swan and white-face whistling duck, and two cases of t15307/ST133/CC133 in another white-face whistling duck and a Baikal teal. This again suggests the potential for exposure to a contaminated environmental source for some of these animals, including water or other shared habitats within the facility.

Other captivity-based studies document the potential for bidirectional transmission between humans and animals in these facilities. In the San Diego zoo, a MRSA outbreak was noted in 2008, with pustules documented on both an elephant calf and three caretakers. Twenty total caretakers were infected over the next month, and the calf was euthanized. Investigation determined that the calf’s infection with MRSA type USA300 likely came from a colonized caretaker, as the other elephants tested were colonization-negative [60].

Isolates examined in collected studies include methicillin-resistant and methicillin -susceptibile *S. aureus*. This testing included a mix of phenotypic and molecular methods, with some studies employing both. With the discovery of *mec*C [11,94], some early papers examining phenotypic testing alone should be looked at with some skepticism, as *mec*C-positive *S. aureus* isolates do not always show up as MRSA phenotypically, which can hinder the detection of *mec*C-carrying isolates [55]. Indeed, wildlife may be a key reservoir for *mec*C, as its presence was noted in a number of European reports (see Table 1). Interestingly, *mec*C has not been reported in any isolates originating in the United States to date.

There are a number of limitations to this review. Sampling was concentrated in a small number of countries and a relatively limited number of animal species have been sampled in different geographic areas, making large-scale comparisons difficult. Sampling techniques and anatomical locations tested within animal species vary among research groups. Most studies employed some sort of live animal swabbing (of noses, throats, skin, cloaca, etc.), but several used feces or scat instead of live animal testing. The studies also differed significantly in molecular and antibiotic resistance testing reported, making generalizations across publications difficult. Access to many animal species is also likely a function of convenience rather than a systematic sampling of all organisms in a particular environment, leading to over-representation of some animals relative to their abundance and an under-representation of others. Additional sampling should be carried out in order to examine the continued evolution of *S. aureus* in wildlife, and to track any strains that may have an increased propensity for zoonotic spread and threat to human health.

## 4. Materials and Methods

### 4.1. Eligibility Criteria

Studies that reported the presence of *S. aureus* (methicillin-resistant or susceptible) in any species of wildlife were eligible for inclusion.

### 4.2. Information Sources and Search Strategies

PubMed, Web of Science, and peer-reviewed materials within ProQuest databases were searched in May 2019 to identify eligible studies. The following search terms were used “MRSA OR Methicillin Resistant *Staphylococcus aureus* OR *Staphylococcus aureus* AND wildlife.” Reference lists of the identified studies were also checked for additional studies. “Wildlife” was defined as wild animals but also captive animals who would typically be wild (such as zoo elephants) and those on nature preserves. Captive animals used as livestock or poultry or otherwise farmed or used as pets or work animals were also excluded. Articles were limited to English language only. Articles were examined and duplicate articles were removed.

Titles and abstracts were examined and articles were retained when there was evidence of *S. aureus* colonization or infection reported within wildlife species as defined above. Citations which included information on *S. aureus* antibiotic resistance and/or molecular typing were included in Table 1 and were grouped by animal species type.

## Figures and Tables

**Table 1:** Summary of wildlife data.

	Animal	Molecular Types Identified	Antibiotic Resistance Identified	Geographic Location	Reference
	(Species or Common Name ^+^)				
**Small mammals**	Norway rat (*Rattus norvegicus*)	t034/ST398	OXA	Canada	[30,31,32,33]
	Norway rat (*Rattus norvegicus*)	t008/ST8; t034/ST398;	AMP, CEF, LEV, MOX, OXA, PEN, Q-D, RIF, TET,	Canada	[34]
		t(new)/ST8;			
		t267/ST97;			
		t002/ST105			
	European rabbit	t843/ST130	PEN, FOX *	Spain	[35]
		t645/ST121, t738/ST121, t741/ST121, t272/ST121, t742/ST425, t745/ST425, t181/ST425	ND	Europe (individual countries not provided)	[36]
	European hedgehog	t386/ST1	PEN, FOX, ERY, CLI^I^, STR	Spain	[35]
	European hedgehog (*Erinaceus europaeus*)	t3256/ST130	OXA *, FOX, BLA	Austria	[37]
	European hedgehog (*Erinaceus europaeus*)	t843/ST130	OXA *	Sweden	[38]
		NT/ST130			
	Hedgehog (*Erinaceus europaeus*)	t843, t10751, t10893, t11015, t3391, t15312, t9111, t978	PEN, CEF *, FOX, OXA, GEN, TET, ERY, CLI, FUS, GEN	Sweden	[39]
	Hedgehog (*Erinaceus europaeus*)	CC130, CC599	MRSA	Germany, Austria	[40]
	Wood mouse (*Apodemus sylvaticus*)	t1535/ST1945	PEN, OXA *, FOX	Spain	[41]
		NT/ST1956	none		
		t9303/ST2328	none		
	Common vole (*Microtus arvalis*)	t120/ST15	PEN	Spain	[41]
		NT/ST1956	none		
		t12365/ST1956	none		
		t12752/ST1956	none		
		t3750/ST2328	none		
		t12363/ST2328	none		
		t12364/ST2766	none		
	Brown rat (*Rattus norvegicus*)	t12863/ST2767		Spain	[41]
	Brown rat (*Rattus norvegicus*)	CC130	MSSA	Germany	[40]
	Yellow-necked mouse	t208/ST49, t4189/ST49, t1773/ST890, t843/ST130	ND	Germany	[42]
	House mouse	t843/ST130	ND	Germany	[42]
	Bank vole	t208/ST49, t4189/ST49	ND	Germany	[42]
	Bank vole (*Myodes glareolus*)	CC49, ST890, ST1959	MSSA	Germany	[40]
	Common vole	t1773/ST890, t15027/ST3252, t3058/ST3252, t3830/ST1956	ND	Germany, Czech Republic	[42]
	Field vole	t1736/ST890, t2311/ST88, t3830/ST1956	ND	Germany	[42]
	Common shrew	t9909/ST3033	ND	Germany	[42]
	Rodents and shrews (various)	ND	OXA, RIF, AMP	Slovakia	[43]
	European marmot (*Marmota marmota*)	CC8, CC30	MSSA	Austria	[40]
	Naked mole rat (*Heterocephalus glaber*)	t084/ST15	PEN, TET	Germany	[44]
	Red squirrel (*Sciurus vulgaris*)	t208/ST49; t307/ST4286; t528/ST4310	PEN, CHL, FQ		[44]
	European beaver (*Castor fiber*)	t3058/ST4614		Germany	[44]
	European pine marten (*Martes martes*), red fox (*Vulpes vulpes*), northern white-breasted hedgehog (*Erinaceus roumanicus*)	t1635/ST8 (MRSA, marten)	AMP, CTX, TET, FOX, ERY, OXA, CLI	Poland	[45]
	European brown hare (*Lepus europaeus*)	t843/ST130	OXA *, FOX, BLA	Germany	[37]
		t10513/ST130	OXA *, FOX, BLA		
	European brown hare (*Lepus europaeus*)	CC5, CC130, CC398, ST2425	MSSA (CC5, ST2425), MRSA (CC130, CC398)	Germany, Sweden	[40]
	European otter (*Lutra lutra*)	t4335/ST2620	OXA *, FOX, BLA	Austria	[37]
	Fox squirrel (*Sciurus niger*)	t1166	None	United States (Iowa)	[46]
	Eastern cottontail rabbit (*Sylvilagus floridanus*)	t008	OXA, TET, ERY	United States (Iowa)	[46]
	European beaver (*Castor fiber*)	t4368/ST1959	none	United States (Iowa)	[46]
	Black-flanked rock wallaby (*Petrogale lateralis*)	CC15	AMP, PEN	Australia	[47]
		CC49	none		
	Yellow-footed rock wallaby (*Petrogale xanthopus*)	CC49	none	Australia	[47]
		CC692			
	Mara (*Dolichotis patagonum*)	t528/ST130	AMP, FOX *	Denmark	[48]
	Mara	t528/ST130, t1166/ST133, t7103/ST133		Denmark	[49]
	Banded mongoose	t084/ST15, t984/ST1		Denmark	[49]
	Capybara	t1166/ST133		Denmark	[49]
	European badger (*Meles meles*)	CC25, ST425	MSSA	Germany, Sweden	[40]
	Red fox (*Vulpes vulpes*)	CC1, CC22, ST425, CC130, CC6, CC7, CC8	MSSA except for CC130 (MRSA)	Germany, Austria, Sweden	[40]
	Lynx (*Lynx lynx*)	CC2767	MSSA	Sweden	[40]
	Wild cat (*Felix silvestris*)	CC49, ST2693	MSSA	Germany	[40]
**Bats**	Straw-colored fruit bat (*Eidolon helvum*)	ST15, ST1725, ST1726, ST1727, ST2463, ST2464, ST2465, ST2466, ST2467, ST2470	PEN, ERY, CLI, CIP, FUS	Nigeria	[50]
	Straw-colored fruit bat (*Eidolon helvum*)	t16686/ST1725, t16693/ST1726, t16697/ST1726, t16701/ST1726, t16703/ST1726, t16704/ST1726, t16733/ST1726, t16696/ST1726, NT/ST3958, t16681/ST3958, t16696/ST3958, t16700/ST3959, t16687/ST3959, t16702/ST3959, t16695/ST4013, t16685/ST4043, t16756/ST4043, t15966/ST4047, t16683/ST3964	TET, PEN	Nigeria	[51]
	Indian flying fox (*Pteropus giganteus*)	t843/ST1245; t15865/ST4288	BLA *	Germany	[44]
	Nathusius pipistrelle (*Pipistrellus nathusii*)	t164/ST389		Germany	[44]
	Egyptian fruit bat	t084/ST15	Not reported	Denmark	[49]
	Egyptian fruit bat (*Rousettus aegyptiacus*)	t15196/ST2984; t15197/ST3259; t15197/ST3301	None	Gabon	[52]
	Peters’s dwarf epauletted fruit bat (*Micropteropus pusillus*)	t15197/ST3302	None	Gabon	[52]
**Large mammals**	Wild boar	t1535/CC130, t7174/CC5,		Spain	[35]
		t1534/CC522, t6386/CC425, t3750/ST2328, t11230/ST2328			
	Wild boar (*Sus scrofa*)	t098/ST1, t127/ST1, t607/ST1, t1401/ST1, t2601/ST1, t11223/ST1, t548/ST5, t2516/ST5, t7174/ST5, t11210/ST5, t11214/ST5, t11219/ST5, t084/ST15, t11218/ST96, t6220/ST130, t3583/ST133, t10476/ST133, t11220/ST133, t189/ST188, t034/ST398, t742/ST425, t6909/ST425, t11222/ST425, t11225/ST425, t11232/ST425, t10712/ST1643, t3750/ST2328, t11227/ST2328, t11230/ST2328, t11229/ST2641, t359/ST2672, t11209/ST2675, t11502/ST2678, t015/ST2681, t6384/ST2682, t011/ST2729	PEN, CHL, TET, STR, TMP	Spain	[53]
	Wild boar (*Sus scrofa*)	t011/ST398, t127/ST1	OXA, TET, ERY, CLI	Spain	[54]
	Wild boar (*Sus scrofa*)	t11212/ST425	PEN, FOX *	Spain	[55]
	Wild boar (*Sus scrofa*)	t3750/ST3220, t1533/ST1, t1533/not identified, t298/not identified, not identified/ST3224, t14312/ST3223, t4311/ST3222, t10668/not identified, t3583/ST133, t3750/not identified, t1230/ST2328, t10712/ST1643, t11230/not identified, t899/ST398, t3750/ST2328, t1533/ST1	PEN, CLI, GEN, FUS, CIP, TET, FOX, OXA, LIN	Portugal	[56]
	Wild boar (*Sus scrofa*)	t127/ST1, t091/ST7, t14149/ST30, t021/ST30, t1773/ST890, t11226/ST3237, t1181/ST3369, t7674/ST425, t12042/ST425, t10855/ST425, t3389/ST425, t15002/ST425, t6092/ST425, t14149/ST425, t15001/ST425, t15000/ST3255, t1181/ST133, t3583/ST133, t742/ST425, t14999/ST425, t571/ST804	AMP, PEN, ERY	Germany	[57]
	Wild boar (*Sus scrofa*)	t6386/ST425, t1181/ST133, t6384/ST133, t6385/ST1643, t6386/ST425, t6782/ST425	None	Germany	[58]
	Wild boar (*Sus scrofa*)	CC59, CC133, ST425, CC9, CC97	MSSA	Germany, Austria	[40]
	Red deer	t1535/CC130, t1125/CC5, NT/ST130, t11225/CC425	PEN	Spain	[35]
	Red deer (*Cervus elaphus*)	t098/ST1, t127/ST1, t11223/ST1, t548/ST5, t11210/ST5, t342/ST30, t2678/ST133, t11215/ST350, t571/ST398, t1077/ST425, t6386/ST425, t6909/ST425, t11208/ST425, t11212/ST425, t11228/ST425, t11231/ST425, t528/ST522, t1534/ST522, t3576/ST522, t742/ST2640, t11211/ST2671, t11226/ST2671, t11233/ST2671, t015/ST2681, t11217/ST2681	PEN, SMX	Spain	[53]
	Red deer (*Cervus elaphus*)	t011/ST398	OXA, TET	Spain	[54]
	Red deer (*Cervus elaphus hispanicus*)	t843/ST1945;	PEN, OXA *, FOX	Spain	[59]
		t1535/ST1945;	PEN, OXA *, FOX		
		t2420/ST133	None		
	Red deer (*Cervus elaphus*)	ST425	MSSA	Germany, Austria	[40]
	Fallow deer	t11212/ST425	PEN, FOX *	Spain	[55]
	European mouflon	t6056/ST133, t11233/ST3237		Spain	[35]
	Mouflon (*Ovis orientalis*)	CC1, CC8	MSSA	Germany, Austria	[40]
	Mongolian sheep (*Ovis ammon f. aries*)	t1773/ST700		Germany	[44]
	Eurasian lynx (*Lynx lynx*)	t032/ST22	BLA, FQ	Germany	[44]
	Roe deer (*Capreolus capreolus*)	t15473/ST425		Germany	[44]
	African elephant (*Loxodonta africana*)	t15467/ST4287		Germany	[44]
	African elephant (*Loxodonta africana*)	USA300	OXA	United States (California)	[60]
	African wildcat (*Felis silvestris lybica*)	t011/ST4289		Germany	[44]
	Iberian ibex (*Capra pyrenaica*)	t002/ST5, t1736/ST130, t3369/ST425, t528/ST581, t843/ST581, t1535/ST581, t3750/ST2328, t11501/ST2328, t11221/ST2637, t7229/ST2639, t11216/ST2639, t528/ST2673	PEN T1773/ST2712, SMX	Spain	[53]
	Iberian ibex (*Capra pyrenaica*)	t011/ST398, t1451/ST398	OXA, TET	Spain	[54]
	Alpine chamois (*Rupicapra r. rupicapra*)	t1523/ST45, t1328/ST22, t1773/ST700	PEN, AMP, AMX, FOX, CIP, FQ, OXA	Italy	[61]
	Chamois (*Rupicapra rupicapra*)	CC133	MSSA	Sweden	[40]
	Roe deer (*Capreolus capreolus*)	t1773/ST2712	None	Italy	[61]
	Roe deer (*Capreolus capreolus*)	ST425, ST133, CC97	MSSA	Germany, Austria, Sweden	[40]
	Silka deer (*Cervus nippon*)	ST3227	MSSA	Germany	[40]
	Fallow deer (*Dama dama*)	CC1, CC130	MSSA (CC1), MRSA (CC130)	Germany	[40]
	Reindeer (*Rangifer tarandus*)	CC707, CC2767	MSSA	Sweden	[40]
	Moose (*Alces alces*)	CC15, CC97, ST2691	MSSA	Sweden	[40]
	Dromedary camel (*Camelus dromedaries*)	ST1755/CC152, CC6, CC30, CC188	ND	United Arab Emirates	[62]
	Malayan tapir	t3583/ST133	Not reported	Denmark	[49]
	Pygmy goat	t304/ST6, t2678/ST133	Not reported	Denmark	[49]
	Lion	t3583/ST133, t7104/ST133, t7355/ST133	Not reported	Denmark	[49]
**Non-human primates**	Rhesus macaque (*Macaca mulatta*)	ST22, ST239	CIP, ERY, GEN, SXT, TET	Nepal	[63]
	Rhesus macaque (*Macaca mulatta*)		OXA, PEN, TET, LVX, CIP	South Korea	[64]
	Rhesus macaque (*Macaca mulatta*)	t189/ST188	PEN, CLI, ERY, GEN, CIP, SXT, MUP	United States (New York)	[65]
		t4167/ST3862	PEN, FOX, GEN, CIP, TET, SXT, MUP		
		t4167/ST3862	PEN, FOX, GEN, CIP, TET, SXT		
		t16708/ST3862	PEN, FOX, GEN, CIP, TET, SXT		
		t16709/ST3862	PEN, FOX, GEN, CIP, SXT		
		t8397/ST3884	None		
	Rhesus macaque	t15469/ST3268	PEN, OXA, TET, CIP	United States (Washington)	[66]
	(*Macaca mulatta*)				

	Japanese macaque (*Macaca fuscata*)	t091/ST7		Germany	[44]
	Barbary macaque (*Macaca sylvanus*)	t091/ST7		Germany	[44]
	Pooled samples from macaque species (*Macaca fascicularis, M. mulatta, M. nemestrina*)	ST188	MRSA: No additional phenotypic testing	United States (Washington)	[67]
		ST3268			
		ST226			
	Southern pig-tailed macaque	t189/ST188	PEN, OXA, ERY, CLI, GEN, KAN, CIP	United States (Washington)	[66]
	(*Macaca nemestrina*)	t189/ST188	PEN, OXA, ERY, CLI, GEN, KAN, TET, CIP, BAC		
		t3887/ST188	PEN, OXA, ERY, CLI, GEN, CAN, CIP		
		t13638/ST3268	PEN, OXA, GEN, KAN, TET, CIP		
		t13638/ST3268	PEN, OXA, GEN, KAN, TET, CIP, BAC		
	Singaporean long-tailed macaque (*Macaca fascicularis*)	t13638/ST3268	PEN, OXA, GEN, KAN, TET, CIP, BAC	United States (Washington)	[66]
	Singaporean long-tailed macaque (*Macaca fascicularis*)	ST3268	CIP, GEN, TET	Singapore	[68]
		ST22	CIP, CLI, ERY		
	Gorilla (*Gorilla gorilla gorilla*)			Cameroon	[69], molecular detection only
	Gorilla (*Gorilla gorilla gorilla*)	t148/ST72	PEN, AMP	Gabon	[70]
	Gorilla (*Gorilla gorilla*)	t6886/ST2074		Gabon	[71]
	Chimpanzee (*Pan troglodytes*)	t148/ST72	PEN, AMP	Gabon	[70]
		t56/ND	Pan-susceptible		
		t5017/ND			
	Chimpanzee	t008, t818, t024, t197, t2030, t9141, t682, t6172 (all USA300/ST8); t116, t1754	Only MRSA collected	United States (Texas)	[72]
	Chimpanzee	t7099/ST188	Not reported	Denmark	[49]
	Chimpanzee (*Pan troglodytes*)	t6962/ST9; t127/ST1; t6963/ST1; t6960/ST601; t7821/ST1782; t6961/ST1856; t6964/ST1928;	^&^	Côte d’Ivoire	[71]
	Chimpanzee (*Pan troglodytes schweinfurthii*)	t934/ST80; t189/ST188; t084/ST2126; t1247/ST2168; t2864/ST2178; t2360/ST6; t355/ST152; t11391/ST1292	TET, PEN, SXT	Uganda	[73]
	Chimpanzee (*Pan troglodytes verus*)	t127/ST1; t1931/ST1; t6963/ST1; t015/ST45; t11388/ST601; t6960/ST601; t6964/ST1928; t11390/ST2603; t11389/ST2621	PEN	Côte d’Ivoire	[73]
	Chimpanzee (*Pan troglodytes*)	t304/ST2020;	PEN	Zambia, Uganda	[74]
		t279/ST15;	PEN, TET		
		t7723/ST15;	PEN		
		t084/ST2126;	PEN		
		t1247/ST2168;	PEN		
		t2864/ST2178;	PEN, ERY, CLI, SXT, TET		
		t934/ST80;	TET		
		t7722/ST101;	PEN, TET		
		t224/ST1948	PEN, TET		
	King colobus (*Colobus polykomos*)	t127/ST1	^&^	Côte d’Ivoire	[71]
	Western red colobus (*Piliocolobus badius*)	t6623/ST2023; t6626/ST2058; NT/ST2059; t6622/ST2072; t6621/NT; t6624/NT; t6625/NT	^&^	Côte d’Ivoire	[71]
	Greater spot-nose monkey (*Cercopithecus nictitans*)	t3636/ST1; t934/ST1855; t6531/ST1854; t6533/ST1872; t6696/ST1873; t6697/ST1874; t7393/ST2067; t6715/ST2071; t6331/NT; t6529/NT; t6747/NT	^&^	Gabon	[71]
	Grey-cheeked mangabey (*Lophocebus albigena*)	t6530/ST1838; t6534/ST1851; t2768/ST1852	^&^	Gabon	[71]
	Gabon talapoin (*Miopithecus ogouensis*)	t6532/ST1853	^&^	Gabon	[71]
	Red-tailed monkey (*Cercopithecus ascanius*)	t6695/ST1857; t6705/ST2022;	^&^	Gabon	[71]
	Moustached guenon (*Cercopithecus cephus*)	t6533/ST1872	^&^	Gabon	[71]
	Mandrillus sp.	t6747/NT	^&^	Gabon	[71]
	Red-fronted lemur (*Eulemur rufifrons*) and Verraux’s sifaka (*Propithecus verreauxi*)	t10694/ST1; t127/ST1; t493/ST182; t189/ST188; t10695/ST2435; t1429/ST2436	PEN	Madagascar	[73]
*Birds*	Cinereous vulture	t011/ST398, t843/ST1945, t843/ST1571, t1535/ST1945, t267/ST97, t5998/ST425	PEN, FOX, ERY, CLI, TET	Spain	[75]
	Magpie	t843/ST1583, t843/ST1945, t843/ST1581	PEN, FOX	Spain	[75]
	Common magpie (*Pica pica*)	CC692	MSSA	Sweden	[40]
	Rook (*Corvus frugilegus*)	CC15, CC88, ST1, ST22	MSSA (CC15, CC88), MRSA (ST1, ST22)	Austria	[40]
	Great tit (*Parus major*)	CC692	MSSA	Sweden	[40]
	Blue-winged teal (*Spatula discors*)	t1535/ST130		Germany	[44]
	Black swan (*Cygnus atratus*)	t1166/ST3269		Germany	[44]
	Mute swan (*Cygnus olor*)	CC133	MSSA	Sweden	[40]
	White-face whistling duck (*Dendrocygna viduata*)	t1166/ST3269; t15307/ST133		Germany	[44]
			TET		
	Baikal teal (*Sibirionetta formosa*)	t15307/ST133			[44]
	White-tailed eagle (*Haliaeetus albicilla*)	t1422/ST692		Germany	[44]
	Golden eagle (*Aquila chrysaetos*)	CC97, CC692	MSSA	Sweden	[40]
	White-tailed eagle (*Haliaeetus albicilla*)	CC692	MSSA	Sweden	[40]
	Red kite (*Milvus milvus*)	t14745/ST692		Germany	[44]
	White stork (*Ciconia ciconia*)	t1818/ST5; t1166/ST133; t6384/ST2682; t6606/ST2377; t571/ST398; t012/ST667; t002/ST5; t688/CC5; t126/CC5; t209/CC5; t045/CC5; t015/ST3060; t843/ST3061; t091/ST7; t011/ST398; t3625/ST398; t774/CC5; t005/CC22; t012/CC30; t216/CC59; t14445/ST22	PEN, TET, CHL, ERY, STR, CLI^I,^ FUS, OXA *, FOX (various isolates)	Spain	[76]
	Common buzzard (*Buteo buteo*)	t012/ST30	PEN, TET, CHL	Portugal	[77]
	Common chaffinch (*Fringilla coelebs*)	t6293	OXA *	Scotland	[78]
	Lesser yellowlegs (*Tringa flavipes*)	t002	OXA, ERY, CLI, LEV	United States (Iowa)	[46]
	Great horned owl (*Bubo virginianus*)	t4735	none	United States (Iowa)	[46]
	Tawny owl (*Strix aluco*)	CC692	MSSA	Sweden	[40]
	Great blue heron (*Ardea herodias*)	t2603	none	United States (Iowa)	[46]
	Rock pigeon (*Columba livia*)	t4634/ST2018	TET, hGISA	United States (Iowa)	[46]
		t1059	none		
	Screech owl (*Megascops* spp.)	t094	TET	United States (Iowa)	[46]
	Eurasian griffon vulture (*Gyps fulvus*)	t7304/ST133	none	Spain	[53]
	Eurasian griffon vulture (*Gyps fulvus*)	t011/ST398	OXA, TET	Spain	[54]
	Grey partridge (*Perdix perdix*)	CC5	MSSA	Sweden	[40]
	Green woodpecker (*Picus viridis*)	CC692	MSSA	Sweden	[40]
	Canada goose (*Branta canadensis*)	t002/ST5;	PEN	United States (Ohio)	[79]
		t688/ST5	PEN		
		too8/ST8;	PEN, OXA, ERY		
		t127/ST8	PEN, OXA, ERY		
		t008/ST8;	PEN		
		t2595/ST8;	PEN, OXA, ERY		
		t1149/ST291;	PEN		
		t1451/ST398;	PEN, ERY, CLI		
		t15031/ST2111	PEN		
**Fish and marine mammals**	Tilapia (*Oreochromis niloticus*)		OXA	Malaysia	[80]
	Dolphin	t002/USA100	OXA	North America	[81]
	Harbour porpoise (*Phocoena phocoena*)	CC12	MSSA	Sweden	[40]
	Walrus	t002/USA100	OXA	North America	[81]

Table 1: Abbreviations: PEN: penicillin; OXA: oxacillin; FOX: cefoxitin; ERY: erythromycin; CLI: clindamycin; STR: streptomycin; KAN: kanamycin; BAC: benzalkonium chloride; RIF: rifampicin; AMP: ampicillin; CIP: ciprofloxacin; GEN: gentamicin; SXT: trimethoprim-sulfamethoxalzole; TMP: trimethoprim; SMX: sulfamethoxazole, LVX: levofloxacin; AMX: amoxicillin; IPM: imipenem; CFZ: cefazolin; FUS: fusidic acid; BLA: β-lactams; CHL: chloramphenicol; Q-D: quinupristin-dalfopristin; FQ: fluroquinolones; hGISA: heterogeneous glycopeptide-intermediate *S. aureus.* I: inducible resistance. *: resistance due to *mecC* gene ND: not determined. NT: non-typeable. ^+^: species name only provided if listed in publication. ^&^ All isolates noted to be susceptible to penicillin, methicillin, aminoglycosides, fluroquinolones, macrolides, lincosamides, nitrofurantoin, Fosfomycin, rifampicin, tetracycline, cotrimoxazole, vancomycin.

## Appendix A

**Table A1:** Picture credits.

Norway Rat: Wikipedia Commons
Wild Boar: Michael Gäbler, CC BY 3.0, https://commons.wikimedia.org/w/index.php?curid=18023043
Iberian Ibex: Juan Lacruz-Own work, CC BY-SA 3.0, https://commons.wikimedia.org/w/index.php?curid=23442996
Cinereous Vulture: Petr Hamerník-Zoo Praha, CC BY-SA 4.0, https://commons.wikimedia.org/w/index.php?curid=81533005
White Stork: Richard Bartz-Own work, CC BY-SA 2.5, https://commons.wikimedia.org/w/index.php?curid=6587475
Eurasian Griffon: Arindam Aditya—Own work, CC BY-SA 4.0, https://commons.wikimedia.org/w/index.php?curid=49792446
European Marten: Green Yoshi-Own work, CC BY-SA 3.0, https://commons.wikimedia.org/w/index.php?curid=20147023
Red Fox: normalityrelief—https://www.flickr.com/photos/normalityrelief/5301299623/in/faves-137790805@N06/, CC BY-SA 2.0, https://commons.wikimedia.org/w/index.php?curid=79116814
European Marmot: Böhringer Friedrich-Own work, CC BY-SA 3.0 at, https://commons.wikimedia.org/w/index.php?curid=22314301
Mouflon: Alexandre Prévot- Flickr: Mouflon de Corse, CC BY-SA 2.0, https://commons.wikimedia.org/w/index.php?curid=21965145
City Hedgehog: Hedera.baltica from Wrocław, Poland—City hedgehog, CC BY-SA 2.0, https://commons.wikimedia.org/w/index.php?curid=43379464
Yellow-Necked Mouse: Donald Hobern from Copenhagen, Denmark—Apodemus flavicollis, CC BY 2.0, https://commons.wikimedia.org/w/index.php?curid=74666734
House Mouse: Bolid74-Own work, CC BY-SA 4.0, https://commons.wikimedia.org/w/index.php?curid=44601165
Fallow Deer: Johann-Nikolaus Andreae-originally posted to Flickr as p9036717.jpg, CC BY-SA 2.0, https://commons.wikimedia.org/w/index.php?curid=46563187
Blue-Winged Teal: Mike’s Birds from Riverside, CA, US-Blue-winged teal, CC BY-SA 2.0, https://commons.wikimedia.org/w/index.php?curid=81471841
European Badger: Kallerna-Own work, CC BY-SA 3.0, https://commons.wikimedia.org/w/index.php?curid=20438656
Roe Deer: Bobspicturebox-Own work, CC BY 3.0, https://commons.wikimedia.org/w/index.php?curid=9384844
Moose: XAlexandraS-Own work, CC BY-SA 4.0, https://commons.wikimedia.org/w/index.php?curid=45694439
Egyptian Fruit Bat: Eggybird-https://www.flickr.com/photos/eggybird/103161513/, CC BY 2.0, https://commons.wikimedia.org/w/index.php?curid=79181194
Capybara: By VigilancePrime at English Wikipedia, CC BY 3.0, https://commons.wikimedia.org/w/index.php?curid=3240452
